# Evolution of Gene Arrangements in the Mitogenomes of Ensifera and Characterization of the Complete Mitogenome of *Schizodactylus jimo*

**DOI:** 10.3390/ijms232012094

**Published:** 2022-10-11

**Authors:** Zhi-Cuo Dan, De-Long Guan, Tao Jiang, Hang Wang, Lu Zhao, Sheng-Quan Xu

**Affiliations:** College of Life Sciences, Shaanxi Normal University, Xi’an 710119, China

**Keywords:** *schizodactyloidea*, mitogenome, long-horned orthotperans, gene arrangements, evolution

## Abstract

Gene arrangement (relative location of genes) is another evolutionary marker of the mitogenome that can provide extensive information on the evolutionary mechanism. To explore the evolution of gene arrangements in the mitogenome of diversified Ensifera, we sequenced the mitogenome of the unique dune cricket species found in China and used it for phylogenetic analysis, in combination with 84 known Ensiferan mitogenomes. The mitogenome of *Schizodactylus jimo* is a 16,428-bp circular molecule that contains 37 genes. We identified eight types of gene arrangement in the 85 ensiferan mitogenomes. The gene location changes (i.e., gene translocation and duplication) were in three gene blocks: I-Q-M-ND2, rrnl-rns-V, and ND3-A-R-N-S-E-F. From the phylogenetic tree, we found that *Schizodactylus jimo* and most other species share a typical and ancient gene arrangement type (Type I), while Grylloidea has two types (Types II and III), and the other five types are rare and scattered in the phylogenetic tree. We deduced that the tandem replication–random loss model is the evolutionary mechanism of gene arrangements in Ensifera. Selection pressure analysis revealed that purifying selection dominated the evolution of the ensiferan mitochondrial genome. This study suggests that most gene rearrangements in the ensiferan mitogenome are rare accidental events.

## 1. Introduction

The mitogenome of insects is a typical circular double-stranded molecule that is 14–20 kb in size and contains 37 genes, including 13 protein-coding genes (PCGs), 22 transfer RNA (tRNA) genes, 2 ribosomal RNA (rRNA) genes, and a sizeable non-coding region called the control region (CR) or A + T-rich region, which has high A + T content [1,2]. Due to its characteristics of conservative gene composition and arrangement, absence (or deficient level) of recombination, uniparental inheritance, and a faster evolutionary rate than that of nuclear DNA, the mitogenome has been extensively used as a standard molecular marker in the phylogenetic, evolutionary, and taxonomic research of animals, especially insects [2,3,4,5].

Gene rearrangement has been extensively reported in various insect mitogenomes [6,7,8,9,10,11,12,13,14,15,16,17,18,19]. The most common type of gene arrangement involves tRNA genes, among which the gene orders in Hymenoptera, Thysanoptera, Psocoptera, Hemiptera, and Phthiraptera exhibit more frequent rearrangements than the putative ancestral gene order [7,20,21,22,23,24,25,26]. Some studies have suggested that mitogenome rearrangement may provide essential clues regarding evolution and origin [2,27,28,29]. In recent years, the discovery of gene rearrangements has dramatically increased with the development of new sequencing methods. Although many mitogenomes have been completely sequenced, only a few gene arrangements have been recorded in Ensifera [30,31].

Ensifera is a group of large and morphologically diverse insect taxa, including crickets, mole crickets, katydids, dune crickets, and wetas. It contains 12,000 species, with approximately 2000 genera in seven superfamilies [32]. Schizodactyloidea is a superfamily of Ensifera insects found in Asia and Africa, composed of only 17 species and two genera (*Schizodactylus* and *Comicus*) [33]. Although they are taxonomically studied, the phylogenetic role of Schizodactyloidea has not been elucidated because of the lack of mitochondrial data and limited molecular research on this lineage [34,35,36]. Schizodactyloidea is considered an ancient group of Orthoptera. Some early morphological studies have suggested that Schizodactyloidea is a sister group to other taxa in the Katydid branch, except for Rhaphidophoroidea. Later, some phylogenetic studies based on molecular data also supported Schizodactyloidea and Tettigonioidea as sister groups [32,36]. In the most recent phylogenetic study of Orthoptera based on mitochondrial genes, Schizodactyloidea was found in the cricket branch [37]. However, the results were obtained using only the mitochondrial genome of the genus *Comicus*.

To further investigate the evolution of the mitochondrial gene order in Schizodactyloidea and compare it with the gene orders of other Ensifera species, we sequenced the complete mitogenome of *Schizodactylus jimo* He 2021, a unique species of Schizodactyloidea that is found in China [38]. We then used the *S. jimo* mitogenome sequence, combined with 84 other available Ensiferan mitogenomes, to carry out a phylogenetic analysis that investigated the types and evolutionary mechanisms of mitochondrial gene arrangements in Ensifera. The results of this study will improve our understanding of the changes in relative gene locations in mitogenomes.

## 2. Results

### 2.1. Mitogenome Features of Schizodactylus jimo

**Figure 1:**
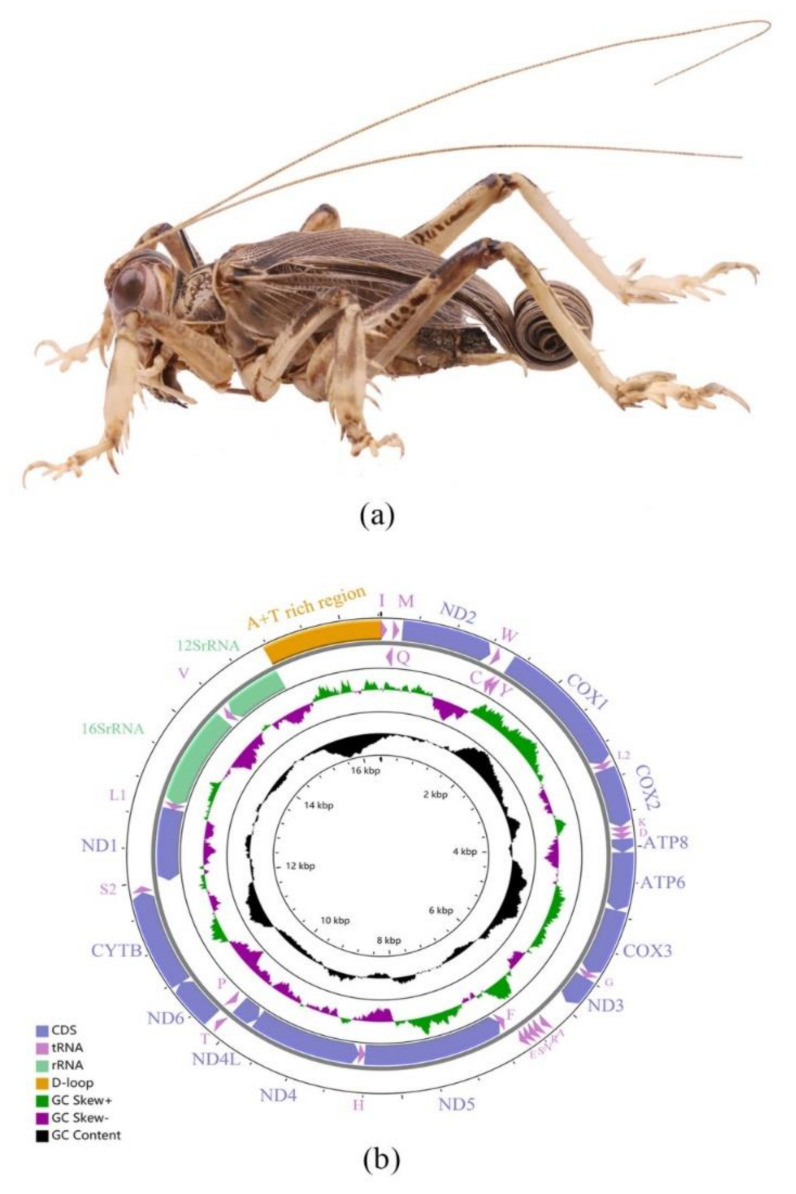
Mitogenome of *S. jimo*. (**a**) Morphology of *S. jimo*, lateral view. (**b**) Circular map of the mitochondrial genome.

**Figure 2:**
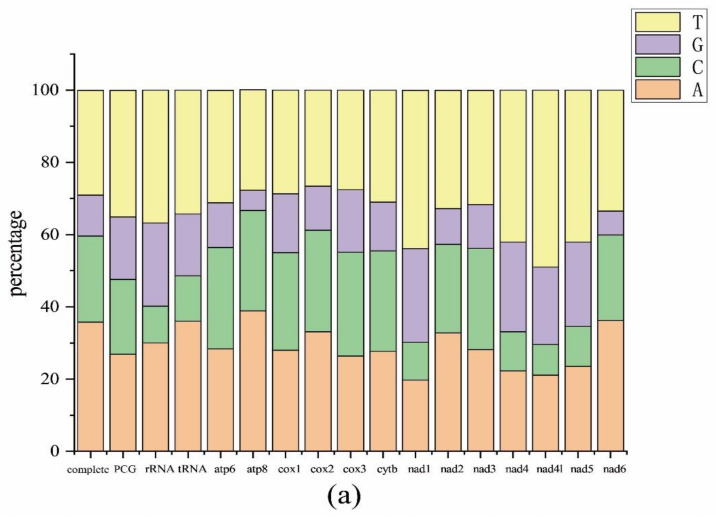

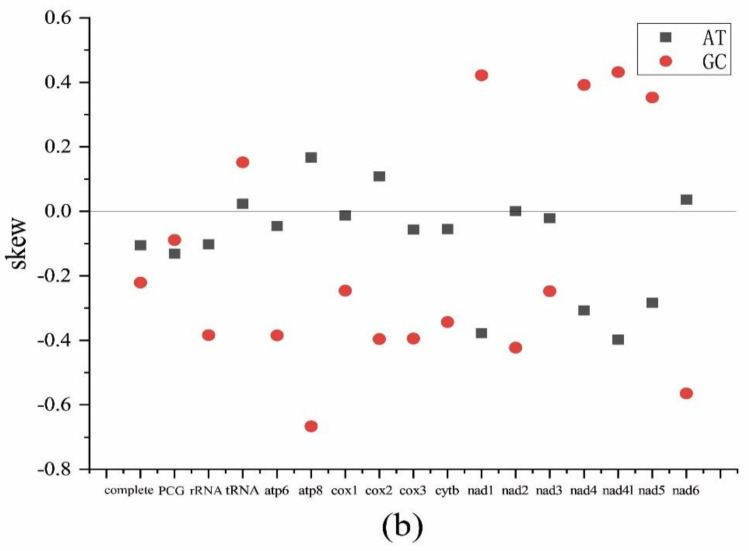
The nucleotide composition and skew of the *S. jimo* mitogenome. (**a**) Nucleotide composition (A/T/G/C percentage) of the mitogenome (indicated as complete) as well as ribosomal RNA (rRNA), transfer RNA (tRNA), and protein-coding genes. (**b**) AT-skew and GC-skew across the mitogenome (indicated as complete) and rRNA, tRNA, and protein-coding genes.

The complete mitogenome of *S. jimo* was 16,428 bp (GenBank ID: OP178893), including 13 PCGs, 22 tRNA genes, 2 rRNA genes, and an A + T rich region (Figure 1). The mitogenome size of S. jimo was in the range of that of the 84 other Ensifera mitogenomes (from 14,971 bp for *Ruspolia dubia* to 18,133 bp for *Sinochlora longifissa*, Table S2). Among these, the light strand (N-strand) encoded 14 genes and the heavy strand (J-strand) encoded 23 genes. The overall AT and GC skews for S. jimo were −0.105 and −0.221, respectively (Figure 2).

**Figure 3:**
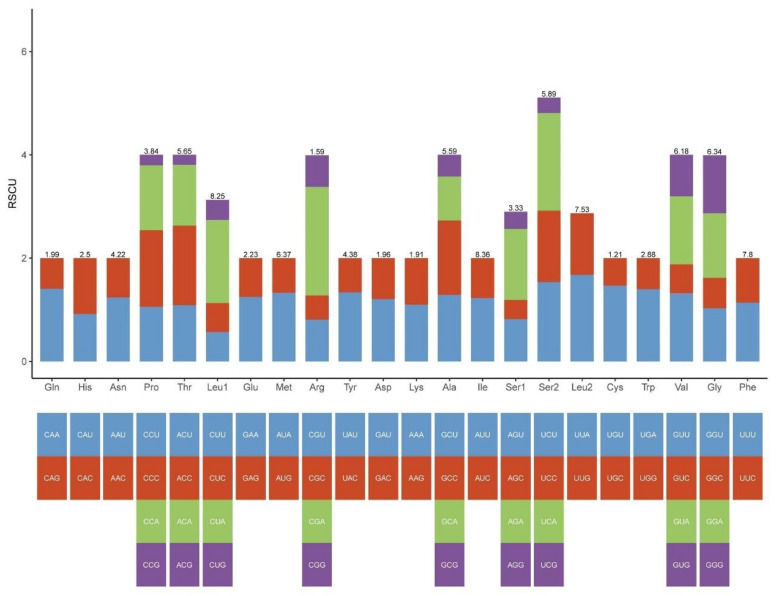
RSCU of the *S. jimoS. jimo* mitogenome. The bar length represents the accumulated scores of all codons for each amino acid (AA). The numbers on each bar represent the AA ratios of all amino acids in the mitochondrial genome.

The total length of the 13 PCGs in *S. jimo* was 11,195 bp, accounting for 68.1% of the entire mitogenome. The mitogenome of *S. jimo* encodes 3731 amino acids (apart from the stop codon). The number of amino acids was within the range of the other Ensifera species (from 3684 in *Capnogryllacris melanocrania* to 3760 in *Orophyllus montanus*, Table S2). Codon usage analysis revealed that the most frequently used codons in the Ensiferan mitogenomes included AUU (for isoleucine; Ile), UUU (for phenylalanine; Phe), and UUA (for leucine; Leu) (Figure 3 and Table S3).

Twenty-two typical tRNA genes were identified in the *S. jimo* mitogenomes, ranging from 63 to 72 bp. All tRNAs could be folded into a typical clover-leaf structure, except for tRNA^Ser^ (AGN), in which the dihydrouridine (DHU) arm formed a loop. The reduced DHU stem of tRNA^Ser^ (AGN) is commonly found in the mitogenomes of other insects, including in all the available mitogenomes of Ensifera. Two rRNA genes (12S and 16S) were identified on the L-strand of the *S. jimo* mitogenome, with the 16S rRNA gene located between tRNA^Leu^ (CUN) and tRNA^Val^, and the 12S rRNA gene present between tRNA^Val^ and the control region. The lengths of the 16S and 12S rRNA genes were 1285 and 765 bp, respectively. The A + T content was 67.7% for the 16S rRNA and 65.2% for the 12S rRNA.

The CR is characterized by high AT content and is thought to regulate mtDNA transcription and replication. The length of the CR of *S. jimo* was 1275 bp, with an AT content of 80.2%. In other Ensiferan species, the CR length ranged from 294 bp (*Mecopoda elongata*) to 2023 bp (*Atlanticus* sp.) (Table S2).

### 2.2. Phylogenetic and Divergence Time Analyses

**Figure 4:**
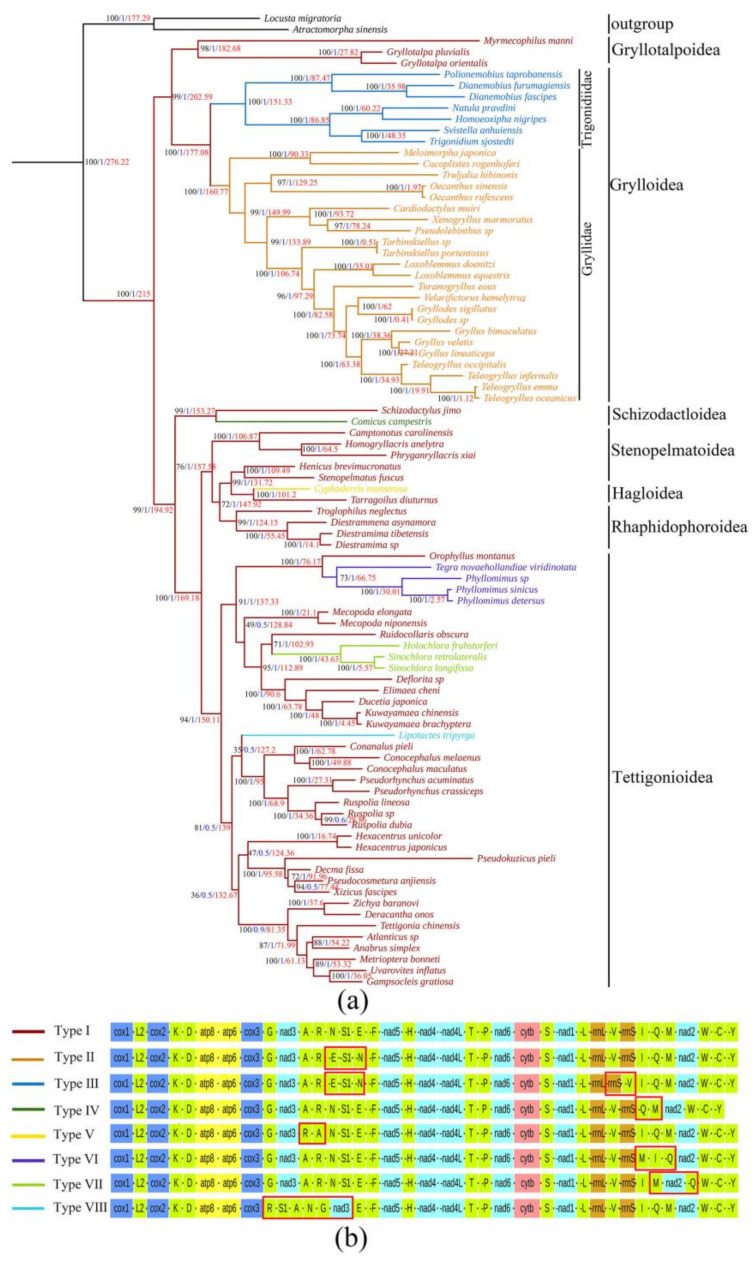
Comparison of mitochondrial gene arrangements among different Ensiferan species. (**a**) The phylogenetic tree was inferred from the nucleotide sequences of 13 PCGs and 2 rRNA genes using Bayesian inference (BI) and maximum likelihood (ML) methods. Values on the branches indicated Bootstrap support. Values are shown next to the nodes; ML bootstrap support values in black, BI bootstrap support values in blue, and divergence times in red. (**b**) Gene orders of the different mitochondrial gene arrangement types.

The ML and BI phylogenetic trees, based on all three codon positions from the 13 PCGs and 2 rRNAs derived from the 85 Ensifera mitogenomes (including the newly sequenced *S. jimo*), were identical (see Figure 4a). Both the ML and BI trees strongly supported the lineage containing the superfamilies Schizodactyloidea, Stenopelmatoidea, Hagloidea, Rhaphidophoroidea, and Tettigonioidea.

*S. jimo* is a sister group to *Comicus campestris*, implying a close relationship between these two lineages. In addition, the clade *S. jimo* + *Comicus campestris* was located at the root of the katydid branch. The relationships between the seven superfamilies of Ensifera were as follows: (Grylloidea, Gryllotalpoidea) (Schizodactyloidea, (Stenopelmatoidea, ((Hagloidea, Rhaphidophoroidea), Tettigonioidea))), consistent with the findings of Zhou et al. [39]. Tettigonioidea is split into two large clades, Tettigoniidae and Phaneropteridae, which are identical to the phylogenetic relationship presented in the Orthoptera Species File Online [40]. To better understand the taxonomic status of Schizodactyloidea and the phylogenetic relationships among Ensifera, there is a need for more sampling of the Schizodactyloidea taxa, which can help in obtaining sufficient molecular data.

Based on the fossil-calibrated phylogeny (Figure 4a), Ensifera originated in the Permian (~276.22 Mya) and diverged into two branches in the Early Triassic period (~215 Mya): a cricket branch and a Katydid branch. The divergence between Grylloidea and Gryllotalpoidea occurred in the Early Triassic period (~202.59 Mya). From the Katydid branch, we dated that *S. jimo* and *Comicus campestris* diverged in the Late Jurassic period (~153.27 Mya). Subsequently, Tettigonioidea and the remaining three superfamilies (Hagloidea, Stenopelmatoidea, and Rhaphidophoroidea) diverged during the middle Jurassic period (~169.18 Mya). The divergence between Rhaphidophoroidea and Hagloidea occurred during the Middle Jurassic period (~147.92 Mya). The divergence between Phaneropteridae and Tettigoniidae occurred during the Middle Jurassic period (~150.11 Mya), and most of the significant subfamilies within Tettigonioidea branched off during the Cretaceous period.

### 2.3. Evolution of Gene Rearrangements

**Figure 5:**
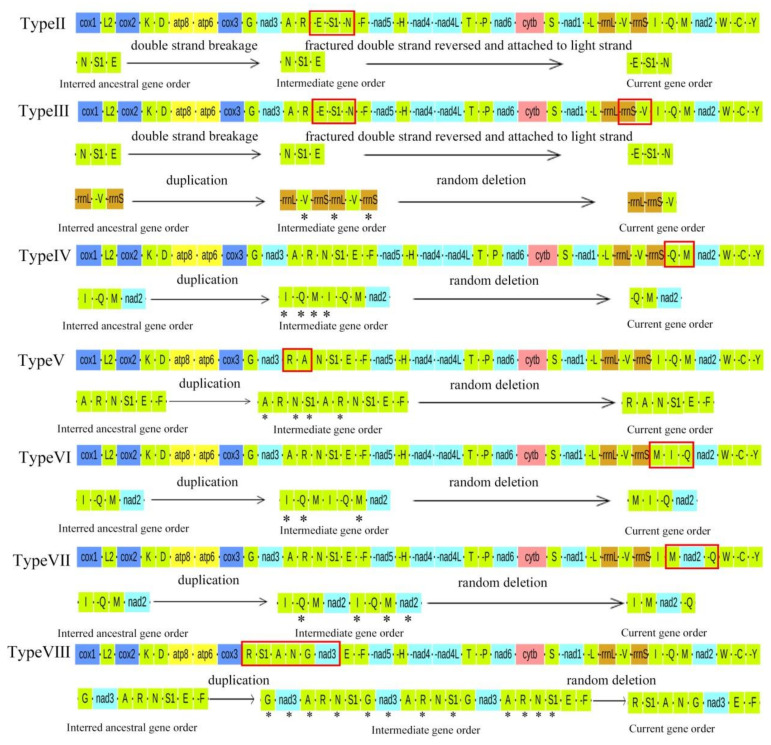
The mechanisms for the eight gene arrangement types in the Ensiferan mitogenome. Genes encoded by the heavy strand (J-strand) have been denoted using a minus sign, whereas those encoded on the light strand (N-strand) have been denoted without one. Genes marked with an asterisk represent ones that were randomly deleted. Differently colored boxes represent different genes.

We identified eight gene arrangement types in Ensifera (Figure 4). Compared to the major Ensifera lineages, the gene order had undergone more significant changes. For example, trnN and trnE of the major Grylloidea lineage were interchanged and moved from heavy to light strands (Type II). However, a unique transposition of rrnS and trnV was found in Trigonidiidae (Type III) compared to the significant mole crickets. The structures of the two dune cricket mitogenomes, *S. jimo* and *Comicus campestris*, were very similar, except for the deletion of trnI in *Comicus campestris* (Type IV). The gene sequence of Tettigonioidea was significantly different from that of most Ensifera species. In *Tegra novaehollandiae viridinotata* and three *Phyllomimus* species, the position of trnM was changed, between rrns and trnI (Type VI). In some Phaneropterinae species, including *Holochlora fruhstorferi* and two *Sinochlora* species, trnQ was inserted after nad2 (Type VII). Compared to the significant Tettigonioidea lineage, more inversion and transpositions were observed in *Lipotactes tripyrga* (Type VIII), trnA and trnR were interchanged, trnS1 was inserted after trnR, and trnG and nad3 were located after trnN. However, gene order changes occurred only in tRNA genes; translocated tRNAs did not change their transcription direction, except for that in the case of Type V (for more details, see Figure 5).

**Figure 6:**
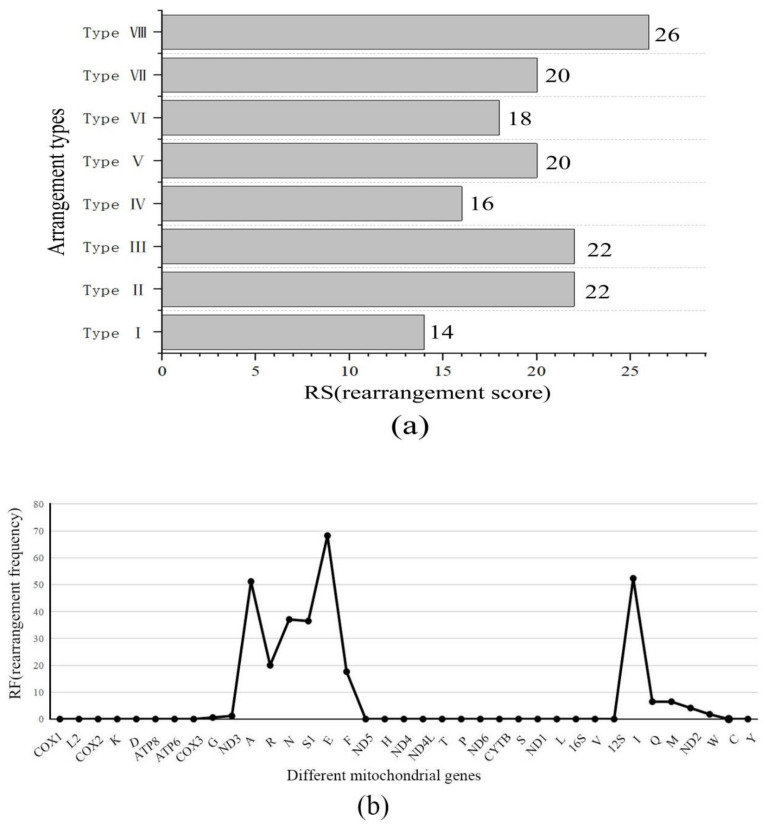
Distribution of rearrangement frequencies (RFs) and rearrangement scores (RSs) for the eight gene arrangement types in Ensifera, based on the application of qMGRs. (**a**) The RS of each arrangement type. (**b**) The RF of every single mitochondrial gene in all the analyzed mitogenomes.

In addition to gene arrangement Type I, which is familiar to most Ensifera spp., the positions of several tRNAs of the other seven types were significantly altered. Type III was the gene arrangement type in Trigonidiidae species, and the positions of one rRNA and several tRNAs changed dramatically, suggesting an overactivated status in mitogenomes of species that underwent Type III rearrangement. We calculated the RS and RF for each gene in all the collected mitogenomes. We calculated the RS of each mitogenome by accumulating the RSs of all genes in the mitogenome. The results indicated that the arrangement Type VIII was a very active group, with the highest RS among Ensifera insects (Figure 6a).

Among the individual genes, tRNA genes had higher RFs than the PCGs (Figure 6b). Among the tRNA genes, A, E, and I exhibited the highest RFs. In addition to tRNA genes, PCGs and rRNA genes were dramatically rearranged in Ensifera.

### 2.4. Evolutionary Analysis of Ensifera Mitogenomes

**Figure 7:**
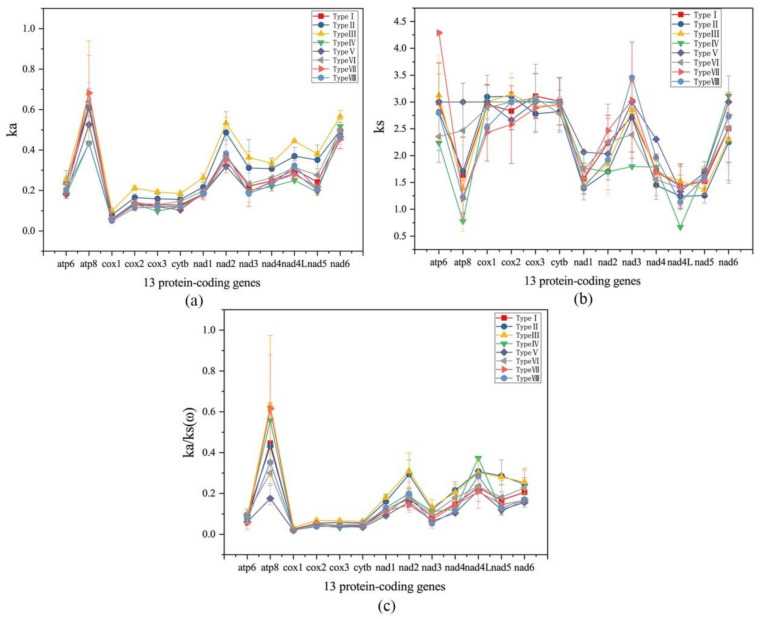
Natural selection strength and the ratio of non-synonymous to synonymous substitutions (ω) were calculated in codeML for the 13 protein-coding mitochondrial genes of the eight Ensifera gene arrangement types. (**a**) ka, (**b**) ks, and (**c**) ka/ks(ω).

Upon examination of the ka and ka/ks values calculated from the 13 PCGs of the 85 Ensiferan mitogenomes, atp8 had the highest averages. This implies that atp8 may have evolved more quickly than the other PCGs in the Ensiferan mitogenomes (Figure 7a,c). Conversely, cox1 had the lowest average ka and ka/ks values (Figure 7a,c). Compared to ka/ks among the 13 mitochondrial PCGs for all insects, all ka/ks(ω) values were lower than 1, indicating that these 13 PCGs were in a state of purification selection (Figure 7).

**Figure 8:**
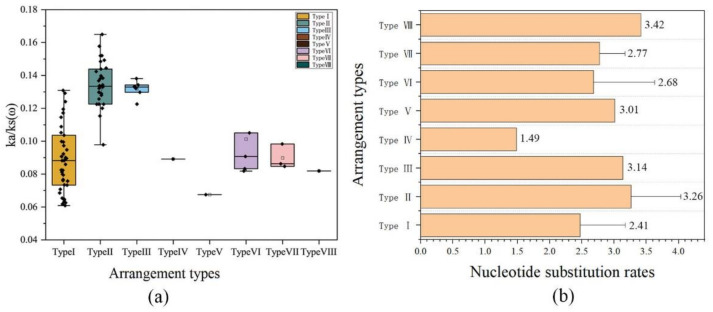
Analysis of natural selection strength and nucleotide substitution rates. (**a**) Histogram of the mean ka/ks of the combined dataset of 13 protein-coding genes. (**b**) Nucleotide substitution rates for the eight gene arrangement types.

Furthermore, all the ka/ks (ω) of the eight gene arrangement types were less than 1, indicating that the eight types were subjected to purification selection (Figure 8a). Type V had the lowest mean value of ω. In contrast, Types II and III had higher mean values of ω. Group comparisons showed no significant differences among the arrangement types with ω values (*p* > 0.05). Type V insects experienced tremendous purification selection pressure. Interestingly, Types II and VII had the highest nucleotide substitution rates, while Type IV had the lowest (Figure 8b).

## 3. Discussion

### 3.1. Evolution of Mitogenomic Gene Rearrangements in Ensifera

Most mitochondrial genomes of Ensifera are in an ancient aligned order, whereas those of Grylloidea species have undergone rearrangement. According to previously reported mitochondrial genomes of Grylloidea, gene rearrangements mainly occurred in the gene blocks A-R-E1-S-N-F and rrnl-rns-V (Type III), while others occurred in A-R-E1-S-N-F (Type II) [41,42,43]. In the Grylloidea species, trnE, trnS (AGN), and trnN are inversed to the minority strand. Therefore, we assumed that the ancestor of Grylloidea possessed the same gene order as ancestral insects. The double-strand breakage of the region “trnN-trnS(AGN)-trnE” would have been the first step, after which the fractured double strand was reconnected to the fracture sites in the minority with the help of enzymes. This is a possible way in which the inversion of the gene cluster “trnE-trnS(AGN)-trnN” could have been formed. The regular rearrangement of Grylloidea could be associated with an adaptation to underground life.

Except for two gene rearrangement types in Grylloidea, all other types are random and irregular. In our study, the mitochondrial gene rearrangements of Ensifera were primarily concentrated in the regions of I-Q-M-ND2 and A-R-N-S-E-F. These gene rearrangements have also been observed in the previously reported mitochondrial genome of Ensifera [42,43,44]. For gene rearrangements of Ensifera, the tandem duplication–random loss model was proposed [45,46,47], which posits that the tandem duplication of mtDNA stretches followed by unexpected loss (via pseudogenization and degeneration) of redundant genes is responsible for between-lineage differences in the mtDNA gene order. This model has already explained similar rearrangements of the gene block CR-I-Q-M in other insects and crabs [48] and even in amphibians [49], which indicates that these are plausible mechanisms that explain the comparable phenomena in Ensifera. The original gene order in this region was assumed to be CR-I-Q-M-ND2. Since the gene rearrangement of *Comicus campestris* was shown as Q-M-ND2, it was inferred as follows: tandem duplication of I-Q-M occurred, followed by accidental loss of trnI.

Consequently, trnI loss was observed in *Comicus campestris*. As for *Holochlora fruhstorferi* and two *Sinochlora* species, their genes were rearranged as I-M-ND2-Q; therefore, we propose a tandem duplication of Q-M-ND2 occurred, followed by accidental loss of the first trnQ and second ND2, trnM. Thus, the genes were arranged as I-M-ND2-Q. In *Tegra novaehollandiae viridinotata* and three *Phyllomimus* species, as compared to the ancestral mitogenome arrangement, trnM was translocated to a position 5′-upstream of trnI, thereby generating the gene order M-I-Q. In Type VII, the gene cluster CR-I-Q-M-ND2 was duplicated, likely promoted by the stem-loop structures detected in the CR. Subsequently, non-random deletions may occur in the entire subset of duplicated genes, depending on the gene copy’s transcriptional polarity and location in the genome, because the CR includes a transcriptional control sequence [50]. Redundant genes that possess the same polarity, such as the redundant gene cluster I-M-ND2, were successively deleted during replication (Figure 4). In contrast, the persistence of trnS (UCN) pseudogenes may have resulted from the tandem duplication of trnS (UCN), followed by a subsequent random loss of trnS (UCN) paralogs.

### 3.2. Selection Constraints on the Mitogenome of Ensifera

All 13 PCGs in the mitogenomes of animals participate in aerobic metabolism. Positive selection is associated with adaptation to new environments. In the 85 mitogenomes studied, the Ka/Ks values for all 13 PCGs were less than 1, suggesting that purifying selection has dominated the evolution of Ensiferan mitogenomes. Among them, the atp8 gene had the highest Ka and Ka/Ks values. In contrast, the cox1 gene had the lowest, indicating that the cox1 gene experienced more substantial evolutionary selection pressure (Figure 7a,b). This phenomenon has been observed in previous studies of insects and fishes [51,52].

Types II and III showed much higher Ka/Ks values among all eight mitogenome types. This indicates that these two types of mitogenomes have higher non-synonymous substitution rates than the others. The higher number of non-synonymous mutations suggests that these cricket species have experienced more relaxed evolutionary constraints, and the slightly beneficial amino acid changes have been fixed in their mitogenomes. Based on the analysis of Ka/Ks ratios and nucleotide substitution rates of all the Ensiferan mitogenomes, we found that mitogenome types with high substitution rates had high RFs. This relationship between the substitution rate and RFs has also been observed in other insect mitogenomes [40,53,54], indicating that there is a need for further studies that investigate the role of nucleotide substitution in mitochondrial gene arrangement in insects.

## 4. Material and Methods

### 4.1. Sample Collection and DNA Extraction

The adult samples of *S. jimo* were collected from Baoshan City, Yunnan Province, China, in 2021. The collected samples were initially placed in 100% ethyl alcohol and stored at −20 °C (College of Life Sciences, Shaanxi Normal University, Xi’an, China). Total DNA was extracted from the whole body of three individuals using a TIANamp Genomic DNA Kit (Tiangen, Beijing, China), following the manufacturer’s instructions. The quality of the DNA samples was checked using 1% agarose gel, and the concentrations were measured using a NanoDrop™ 2000 spectrophotometer (Thermo Fisher, Waltham, MA, USA).

### 4.2. Mitogenome Assembly, Annotation, and Analysis

The entire mitochondrial genome was sequenced using a HiSeq 2500 platform (Illumina, San Diego, CA, USA) by Biomarker Biotechnologies Inc., Beijing, China. The mitogenome of *S. jimo* was assembled using the GetOrganell pipeline v1.7.5.3 [55], in which KM657337 was used as the initial bait to screen for mitochondrial reads. Following the spades, a Denovo assembly software was used to construct these reads into mitogenomic contigs. The 13 PCGs were predicted through comparison with the homologous sequence of reference mitogenomes and by finding the open reading frames based on the invertebrate mitochondrial genetic code. The locations of 22 tRNAs were identified using MITOS WebServer (http://mitos.bioinf.uni-leipzig.de/index.py, 2012, accessed on 20 October 2021) [56]. The two rRNA genes (rrnS and rrnL) and the A + T-rich region were determined based on the locations of adjacent genes (trnL1 and trnV) and alignment with homologous sequences of reference mitogenomes.

The nucleotide composition and skew, codon usage of PCGs, and relative synonymous codon usage values of each PCG were calculated using PhyloSuite v1.2.1 [57]. At the same time, tandem repeat units of the A + T-control region were analyzed using the Tandem Repeats Finder online server (http://tandem.bu.edu/trf/trf.html, 1999, accessed on 21 October 2021) [58]. Differences in the nucleotide composition (composition skew) were measured using the following formulae: AT-skew = [(A − T)/(A + T)] and GC-skew = [(G − C)/(G + C)] [59]. Evolutionary rate analysis of genes was performed using Phylogenetic Analysis by Maximum Likelihood (PAML) v4.9 [60]. The maps of mitochondrial DNA (mtDNA) were drawn using CGView Server v1.0 [61].

### 4.3. Phylogenetic Analyses and Estimation of Divergence Time

We prepared a dataset comprising the novel mitogenome of *S. jimo* and the complete mitogenomes of 84 other insect species downloaded from NCBI GenBank (see Tables S1 and S2), using two grasshopper species [*Atractomorpha sinensis* and *Locusta migratoria* (Acrididae)], as outgroup taxa. We performed phylogenetic analyses on this dataset using Bayesian inference (BI) and maximum likelihood (ML) methods. Nucleotide sequences were aligned using the G-INS-I (accurate) strategy and codon alignment mode, while amino acid sequences were aligned using auto strategy and the normal alignment mode. We aligned the nucleotide and amino acid sequences of 13 PCGs from each mitogenome using the multiple sequence alignment program MAFFT in PhyloSuite v1.2.1 [57]. The optimal partitioning scheme and nucleotide substitution model for BI and ML phylogenetic analyses with PartitionFinder-2.1.1 were incorporated into PhyloSuite v1.2.1 [62].

We used the Penalized likelihood (PL) method with a truncated Newton algorithm in R8S v1.81 [63] to estimate divergence times, using an input tree with the consensus topology and branch lengths from MrBayes [64]. Five known divergence times were used as calibration points (*Gampsocleis gratiosa* with *A nabrus simplex*: 57 Mya [37]; *Conocephalus maculatus* with *Ruspolia dubia*: 95 Mya [37]; *Ruspolia lineosa* with *Xizicus fascipes*: 139 Mya [37]; *Elimaea cheni* with *Mecopoda elongate*: 128 Mya [37]; and *Myrmecophilus manni* with *Gryllodes* sp: 215 Mya [37]).

### 4.4. Gene Arrangement Analysis in the Mitogenome

Gene orders were extracted using the PhyloSuite software package and visualized on the Interactive Tree of Life (iTOL) website server (https://itol.embl.de/, accessed on 11 November 2021) [65,66]. For a quantitative comparison of gene arrangements in each mitogenome, we calculated the rearrangement score (RS) and rearrangement frequency (RF) of each gene in all collected mitogenomes using the quantifying mitochondrial genome rearrangement (qMGR) approach [67]. The RS of each mitogenome can be calculated by accumulating the RSs of all genes in one genome; this value can be further used as a quantitative feature of mitogenome gene rearrangements.

### 4.5. Analysis of Natural Selection Strength and Nucleotide Substitution Rates

To evaluate the potential adaptive evolution of the mitochondrial genes in Ensifera insects, we performed positive selection analyses using PAML. Synonymous substitutions in protein-coding sequences cannot cause changes in amino acids, typically found in the third position or sometimes in the first position of a codon. Thus, we used a gene-level approach based on the ratio of non-synonymous (Ka) to synonymous (Ks) substitution rates to detect potential positive selection signals on PCGs across closely related or divergent species. We divided the 85 species into eight groups, according to their arrangement types, to explore whether there were differences between different arrangement types under natural selection pressure.

We estimated the mean nucleotide substitution rate using R8S to determine the major driving forces of mitochondrial evolution [63].

## 5. Conclusions

Based on sequencing the mitogenome of *S. jimo*, a unique dune cricket in China, and phylogenetic analysis of known mitogenomes, we identified eight types of gene arrangements in Ensiferan mitogenomes. Crickets (Grylloidea) have two mitogenomic types, which have experienced relaxed evolutionary constraints. Most species share a common ancient gene arrangement type. The remaining types are randomly distributed in the phylogenetic tree. These gene rearrangements can be explained using the tandem replication–random loss model. Mitogenome types with high substitution rates have high RFs, but further studies are needed to investigate the role of nucleotide substitution in mitochondrial gene arrangement in insects.

## Data Availability

The data are contained within the article or Supplementary Materials.

## Supplementary Materials

The following supporting information can be downloaded at: https://www.mdpi.com/article/10.3390/ijms232012094/s1.
