# Supplementary material for: Evolution of Gene Arrangements in the Mitogenomes of Ensifera and Characterization of the Complete Mitogenome of Schizodactylus jimo
Source: Int J Mol Sci. 2022 Oct 11;23(20):12094. doi: 10.3390/ijms232012094 (PMC9603354; doi:10.3390/ijms232012094)
Supplement: Supplementary file 1 [file ijms-23-12094-s001.zip › ijms-1929675-supplementary.pdf]

Table S1 List of taxa used for the phylogenetic analyses in this study

Taxa	Mitogenome size(bp)	GenBank accession no.	Reference	Species distributions	Sample Locality
Gryllidae					
Gryllinae					
Loxoblemmus doenitzi	15,396	KX057721	Zhou *et al.* 2017	Africa, Asia and Australia	German
Teleogryllus emma	15,660	EU557269	Ye *et al.* 2008	China and Japan	German
Teleogryllus oceanicus	15,660	KT824636	Li *et al.*, unpublished	Australasia, Asia-Temperats	Canberra
Tarbinskiellus portentosus	15,498	MZ427921	Wang *et al.* 2022	China	London
Teleogryllus occipitalis	15,501	MZ440652	Unpublished	Asia-Temperats, Asia_Tropical	London
Teleogryllus infernalis	15,512	MK903574	Unpublished	China and Japan	Vienna Museum
Tarbinskiellus sp.	15,514	MZ440655	Unpublished	Asia-Temperats, Asia_Tropical	Lost
Gryllodes sp.	15,550	MZ440657	Unpublished	Australia, Asia, Africa, central Europe, subtropical and tropical Americas	Lost
Gryllus lineaticeps	15,607	NC057052	Unpublished	Northern America	Nroth America
Gryllus veletis	15,686	NC057053	Unpublished	Northern America	Ann Arbor
Turanogryllus eous	16,045	MK656322	Unpublished	Asia-Temperate,China	Petersburg
Gryllus bimaculatus	16,075	NC053546	Unpublished	Europe，Africa，Asia-Temperats	Philadelphia
Velarifictorus hemelytrus	16,123	NC030762	Unpublished	Asia_Tropical	Vienna Museum
Loxoblemmus equestris	16,314	KU562919	Unpublished	Asia-Temperats, Asia_Tropical	Vienna Museum
Gryllodes sigillatus	16,396	NC057195	Unpublished	China, Japan, North Korea, India, Pakistan, Malaysia, Mexico, South America, Australia	London
Eneopterinae					
Xenogryllus marmoratus	15,762	NC041236	Unpublished	Asia-Temperats, Asia_Tropical	Paris
Pseudolebinthus sp.	16,075	MN414243	Unpublished	Africa	
Cardiodactylus muiri	16,328	NC037914	Unpublished	Asia_Tropical	Paris
Podoscirtinae					

Truljalia hibinonis	15,120	NC034797	Li *et al.* 2018	Asia-Temperats	Sapporo
Oecanthinae					
Oecanthus rufescens	15,617	KX057720	Zhou *et al.* 2017	Australasia, Pacific, Asia-Temperats，Asia_Tropical	Paris
Oecanthus sinensis	16,142	NC034799	Li *et al.* 2018	China	London
Trigonidiidae					
Trigonidiinae					
Homoeoxipha nigripes	15,679	NC045841	Lu 2018	China	Shanghai
Trigonidium sjostedti	15,763	NC032077	Ma *et al.* 2018	Australasia	Stockholm
Svistella anhuiensis	16,949	NC053543	Unpublished	China	Shanghai
Dianemobius furumagiensis	15,350	NC045847	Unpublished	China and Japan	Tokyo
Dianemobius fascipes	15,363	NC045846	Unpublished	China and Japan	London
Natula pravdini	15,817	NC050742	Unpublished	China	Petersburg
Nemobiinae					
Polionemobius taprobanensis	16,641	NC045848	Unpublished	Sri Lanka, China and Japan	London
Phalangopsidae					
Cachoplistinae					
Meloimorpha japonica	15,880	NC039665	Ma *et al.* 2018	China and Japan	Leiden
Cacoplistes rogenhoferi	16,081	NC039664	Ma *et al.* 2018	Asia-Temperats, Asia_Tropical	Vienna
Gryllotalpidae					
Gryllotalpinae					
Gryllotalpa orientalis	15,521	AY660929	Kim *et al.* 2005	Asia-Temperats, Asia_Tropical	Madrid
Gryllotalpa pluvialis	15,525	EU938371	Fenn *et al.* 2008	Australasia	Stockholm
Myrmecophilidae					
Myrmecophilinae					
Myrmecophilus manni	15,323	EU938370	Fenn *et al.* 2008	Northern America	Schimmer
Prophalangopsidae					
Cyphoderrinae					
Cyphoderris monstrosa	16,590	KM657332	Song *et al.* 2015	Northern America	Harvard
Prophalangopsinae					

Tarragoilus diuturnus	16,144	JQ999995	Zhou *et al.* 2014, 2017	China	Petersburg
Anostostomatidae					
Anostostomatinae					
Henicus brevimucronatus	15,140	KM657338	Song *et al.* 2015	Africa	Geneva
Gryllacrididae					
Gryllacridinae					
Camptonotus carolinensis	15,211	KM657333	Song *et al.* 2015	Northern America	German
Homogryllacris anelytra	15,706	KX057738	Zhou *et al.* 2017	China	Hebei
Phryganogryllacris xiai	15,876	KX057734	Zhou *et al.* 2017	China	Shanghai
Stenopelmatidae					
Stenopelmatinae					
Stenopelmatus fuscus	15,767	KM657331	Song *et al.* 2015	Northern America, Southern America, Africa	Lost
Tettigoniidae					
Bradyporinae					
Deracantha onos	15,650	EU137664	Zhou *et al.* 2009	China	German
Zichya barabovi	15,645	KX057716	Zhou *et al.* 2017	China	Petersburg
Conocephalinae					
Conanalus pieli	15,820	KX057724	Zhou *et al.* 2017	China	Shanghai
Conocephalus maculatus	15,898	HQ711931	Zhou *et al.* 2011,2017	China	London
Conocephalus melaenus	15,852	KX057725	Zhou *et al.* 2017	China	Leiden
Pseudorhynchus acuminatus	16,056	KX057732	Zhou *et al.* 2017	China	German
Pseudorhynchus crassiceps	15,865	KX057728	Zhou *et al.* 2017	China	Leiden
Ruspolia dubia	14,971	EF583824	Zhou *et al.* 2007,2017	China	Lost

Ruspolia lineosa	16,110	KX057729	Zhou *et al.* 2017	China	London
Ruspolia sp.	15,802	KX057717	Zhou *et al.* 2017	China	Lost
Hexacentrinae					
Hexacentrus japonicus	16,120	KX057713	Zhou *et al.* 2017	China	Vienna
Hexacentrus unicolor	15,752	KX057739	Zhou *et al.* 2017	China	Gernan
Lipotactinae					
Lipotactes tripyrga	15,949	KX057736	Zhou *et al.* 2017	China	Hebei
Meconematinae					
Decma fissa	16,122	KX057710	Zhou *et al.* 2017	China	Hunan
Pseudocosmetura anjiensis	16,044	KX057711	Zhou *et al.* 2017	China	Zhejiang
Pseudokuzicus pieli	16,077	KX057712	Zhou *et al.* 2017	China	Shanghai
Xizicus fascipes	16,166	JQ326212	Yang *et al.* 2012, Zhou *et al.* 2017	China	Chinese Academy of Sciences
Tettigoniinae					
Anabrus simplex	15,766	EF373911	Fenn *et al.* 2007	Northern America	Philadelphia
Atlanticus sp.	16,788	KX057730	Zhou *et al.* 2017	China	Lost
Chizuella bonneti	16,256	KX057723	Zhou *et al.* 2017	China	China
Gampsocleis gratiosa	15,929	EU527333	Zhou *et al.* 2008, 2017	China	German
Tettigonia chinensis	16,244	KX057727	Zhou *et al.* 2017	China	German
Uvarovites inflatus	15,956	KP098593	Zhao, unpublished	China	Vienna
Mecopodinae					

Mecopoda elongata	15,284	JQ917910	Zhou *et al.* 2013, 2017	China	London
Mecopoda niponensis	15,364	JQ917909	Zhou *et al.* 2013, 2017	China	Leiden
Phaneropterinae					
Deflorita sp.	16,831	KX057719	Zhou *et al.* 2017	China	Lost
Ducetia japonica	16,276	KU885974	Zhou *et al.* 2017	China	London
Elimaea cheni	15,831	GU323362	Zhou *et al.* 2010, 2017	China	Beijing
Holochlora fruhstorferi	15,899	KX057733	Zhou *et al.* 2017	China	Geneva
Kuwayamaea brachyptera	16,237	KT345950	Yang *et al.* 2016	China	Chinese Academy of Sciences
Kuwayamaea chinensis	15,875	KX057735	Zhou *et al.* 2017	China	Petersburg
Ruidocollaris obscura	16,424	KT345952	Yang *et al.* 2016	China	Shanghai
Sinochlora longifissa	18,133	KC467055	Liu *et al.* 2013	China and Japan	Sapporo
Sinochlora retrolateralis	17,209	KC467056	Liu *et al.* 2013	China	Fujian
Pseudophyllinae					
Orophyllus montanus	17,015	KX057714	Zhou *et al.* 2017	China	Bonn
Phyllomimus detersus	16,007	KT345949	Yang *et al.* 2016	Asia-Temperats, Asia_Tropical	London
Phyllomimus sinicus	15,692	KX057737	Zhou *et al.* 2017	China	German
Phyllomimus sp.	16,298	KX057722	Zhou *et al.* 2017	China	Lost
Tegra novaehollandiae viridinotata	16,376	KX057715	Zhou *et al.* 2017	China	German
Rhaphidophoridae					
Aemodogryllinae					

Apteranabropsis sp.	16,060	KX057740	Zhou *et al.* 2017	China	Lost
Diestrammena asynamora	15,309	KX057726	Zhou *et al.* 2017	China	Lost
Diestramima sp.	16,346	KX057718	Zhou *et al.* 2017	China	Lost
Troglophilinae					
Troglophilus neglectus	15,810	EU938374	Fenn *et al.* 2008	Europe	German
Schizodactylidae					
Comicinae					
Comicus campestris	15,691	KM657337	Song *et al.* 2015	Africa	Windhoek
Schizodactylinae					
Schizodactylus jimo	16,428	OP178893	This study	Asia-Temperats, Asia_Tropical	China
Pyrgomorphidae					
Pyrgomorphinae					
Atractomorpha sinensis	15,558	EU263919	Ding *et al.* 2007	China and Japan	Paris
Acrididae					
Oedipodinae					
Locusta migratoria	15,722	X80245	Flook *et al.* 1995	Europe, Africa, Asia-Temperats, Asia_Tropical	London

TableS2 Summarized mitogenomic characteristics of the 85 Ensifera species investigated in this study

Species	Whole genome			PCGs				16S rRNA		12S rRNA		A+T-rich	
	Size(bp)	A+T(%)	AT-skew	Size(bp)	No.of codons	A+T(%)	AT-skew	Size(bp)	A+T(%)	Size(bp)	A+T(%)	Size(bp)	A+T(%)
Loxoblemmus doenitzi	15,396	73.3	-0.045	11,193	3730	72.3	-0.132	1296	75.7	757	71.5	663	77.6
Teleogryllus emma	15,660	73.1	-0.108	11,128	3760	72.7	-0.144	1293	75.3	812	71.7	940	73.9
*Teleogryllus oceanicus**	15,660	73	-0.107	11,141	3712	72.5	-0.147	1311	75.1	813	71.6		
Oecanthus rufescens	15,617	76.9	-0.025	11,138	3711	77	-0.124	1300	798	744	77.1	877	68.3
Gryllotalpa orientalis	15,521	70.5	-0.045	11,136	3708	69.5	-0.161	1247	72.8	719	79.4	920	74.9
*Gryllotalpa pluvialis**	15,525	72.2	-0.042	11,090	3694	71.1	-0.158	1236	74.9	783	72.1		
*Myrmecophilus manni**	15,323	70.2	-0.068	11,059	3684	69	-0.136	1252	74.6	734	79.7		
Cyphoderris monstrosa	16,590	71.5	-0.11	11,206	3734	69.7	-0.116	1329	75.3	796	72.7	1188	80.1
Tarragoilus diuturnus	16,144	67.2	-0.079	11,223	3739	66.1	-0.141	1340	71.8	783	76.6	1301	66.2
Henicus brevimucronatus	15,140	73.3	-0.059	11,177	3725	72.2	-0.151	1334	76.5	788	73	172	82.6
Camptonotus carolinensis	15,211	69.3	-0.072	11,165	3719	68	-0.159	1286	73.5	772	71.2	473	68.2
Homogryllacris anelytra	15,706	70.9	-0.073	11,217	3738	69.7	-0.145	1294	74.5	768	71	978	74.6
Phryganogryllacris xiai	15,876	72.3	-0.065	11,227	3742	71.2	-0.138	1303	74.9	762	72.9	1101	75.9
Stenopelmatus fuscus	15,767	70.3	-0.059	11,207	3733	69.3	-0.146	1324	75.6	781	72.7	916	69.2
Deracantha onos	15,650	69.3	-0.036	11,210	3734	67.8	-0.147	1301	72.2	858	70.1	815	77.8
Zichya barabovi	15,645	71.7	-0.029	11,208	3734	70.7	-0.159	1310	74.5	779	70.7	889	78.7
Conanalus pieli	15,820	74.3	-0.029	11,207	3734	73.1	-0.130	1328	77.4	787	75	959	81.2
Conocephalus maculatus	15,898	72.1	-0.043	11,215	3737	70.9	-0.153	1305	75.3	836	71.6	662	79
Conocephalus melaenus	15,852	71.5	-0.037	11,213	3736	70	-0.158	1317	75.9	782	71.8	709	79.4
Pseudorhynchus acuminatus	16,056	71.7	-0.062	11,219	3738	70.2	-0.146	1346	75.2	801	71.4	1113	79

Pseudorhynchus crassiceps	15,865	72.6	-0.059	11,222	3739	71.1	-0.141	1327	75.6	799	73.3	1007	78.7
*Ruspolia dubia**	14,971	70.8	-0.023	11,216	3737	69.9	-0.164	1302	74.4	882	72.2		
Ruspolia lineosa	16,110	71.2	-0.026	11,214	3737	69.8	-0.161	1323	76.1	818	72.7	1235	75.0
Ruspolia sp.	15,802	71.4	-0.039	11,215	3739	70.2	-0.150	1314	74.8	809	72	985	78.5
Hexacentrus japonicus	16,120	69.3	-0.048	11,227	3740	67.7	-0.144	1305	74.2	783	71.3	1307	72.5
Hexacentrus unicolor	15,752	70.0	-0.046	11,221	3739	68.4	-0.143	1306	74.5	781	71.3	929	76.3
Lipotactes tripyrga	15,949	72.2	-0.030	11,249	3749	71.3	-0.121	1315	77.2	829	73.4	917	77.1
Decma fissa	16,122	74.9	-0.033	11,215	3737	73.1	-0.154	1311	78.1	789	74.6	1338	76.6
Pseudocosmetura anjiensis	16,044	71.3	-0.040	11,218	3738	69.6	-0.155	1304	75.2	787	73.7	1241	76.0
Pseudokuzicus pieli	16,077	73.4	-0.025	11,221	3739	73.0	-0.150	1311	77.7	787	73.7	1294	69.3
Xizicus fascipes	16,166	70.2	-0.052	11,221	3739	79.5	-0.156	1307	74.5	790	73.1	1378	65.5
Anabrus simplex	15,766	69.5	-0.030	11,198	3730	67.7	-0.170	1312	72.8	785	68.9	987	80.1
Atlanticus sp.	16,788	67.8	-0.042	11,222	3739	66.7	-0.159	1309	72.4	774	68.5	2023	67.7
Chizuella bonneti	16,256	67.8	-0.031	11,228	3741	67.5	-0.167	1310	72.3	781	70.2	1473	60.0
Gampsocleis gratiosa	15,929	65.3	-0.062	11,212	3735	63.7	-0.158	1317	70.0	848	67.7	1111	67.4
Tettigonia chinensis	16,244	70.4	-0.054	11,233	3743	69.9	-0.142	1346	74.1	668	68.7	731	65.7
Uvarovites inflatus	15,956	67	-0.043	11,218	3737	65.4	-0.171	1317	71.0	846	68.9	1127	71.6
Mecopoda elongata	15,284	71.8	-0.009	11,239	3745	71.2	-0.161	1334	75.4	787	71.5	294	71.5
Mecopoda niponensis	15,364	72.4	-0.006	11,240	3745	71.8	-0.164	1338	75.5	787	71.9	393	69.5
Deflorita sp.	16,831	69.8	-0.023	11,194	3730	69.1	-0.146	1315	73.3	777	71.9	961	62.3
Ducetia japonica	16,276	74	-0.010	11,215	3737	73.1	-0.163	1308	77.0	786	73.0	868	80.0
Elimaea cheni	15,831	72.6	-0.021	11,224	3740	71.6	-0.151	1311	76.3	828	74.5	999	75.4

Holochlora fruhstorferi	15,899	70.2	-0.003	11,181	3726	68.7	-0.156	1305	73.8	779	73.1	1012	72.4
Kuwayamaea brachyptera	16,237	71.6	-0.027	11,212	3736	70.9	-0.148	1308	75.1	808	73.0	804	80.9
Kuwayamaea chinensis	15,875	72.0	-0.025	11,208	3735	70.7	-0.151	1308	75.2	780	72.6	855	81.4
Ruidocollaris obscura	16,424	73.5	-0.011	11,196	3730	72.1	-0.161	1302	76.4	829	72.9	970	76.2
*Sinochlora longifissa**	18,133	69.1	-0.002	11,173	3722	68.9	-0.146	1303	73.3	783	70.8		
Sinochlora retrolateralis	17,209	70.1	-0.006	11,170	3720	69.1	-0.145	1300	73.8	781	71.6	1386	75.6
Orophyllus montanus	17,015	68.1	-0.069	11,262	3753	66.4	-0.139	1338	71.3	782	71.0	892	61.9
Phyllomimus detersus	16,007	71.7	-0.052	11,257	3752	70.4	-0.124	1334	74.4	833	71.6	890	77.7
Phyllomimus sinicus	15,692	71.9	-0.052	11,235	3744	70.5	-0.121	1329	74.6	787	72.1	650	81.6
Phyllomimus sp.	16,298	72.7	-0.054	11,252	3750	72.4	-0.126	1347	76.3	787	72.7	1175	66.2
Tegra novaehollandiae viridinotata	16,376	73.0	-0.045	11,249	3749	72.3	-0.116	1336	78.1	787	70.4	835	62.7
Diestramima tibetensis	16,060	76.7	-0.057	11,229	3742	75.5	-0.129	1332	79.7	791	76.1	1172	74.7
Diestrammena asynamora	15,309	75.0	-0.101	11,229	3742	74.2	-0.128	1356	79.1	790	76.3	382	77.2
Diestramima sp.	16,346	75.7	-0.053	11,217	3738	74.2	-0.136	1334	78.9	791	75.2	1446	83.8
*Troglophilus neglectus**	15,810	73.4	-0.011	11,209	3734	72.6	-0.157	1342	76.9	785	73.1		
Comicus campestris	15,691	75.0	-0.015	11,256	3751	73.7	-0.137	1287	77.0	778	75.4	628	82.3
Schizodactylus jimo	16,428	64.8	-0.105	11,195	3731	61.9	-0.131	1285	67.7	765	65.2	1275	80.2
Natula pravdini	15,817	78.8	-0.01	11,163	3718	77.7	-0.196	1395	79.1	754	79.7	710	88.5
Oecanthus sinensis	16,142	74.4	-0.021	11,126	3707	66.9	-0.194	1298	79.8	747	77.1	1448	78.4
Polionemobius taprobanensis	16,641	70.3	0.015	11,104	3698	69.5	-0.228	1273	73.8	753	71.3	516	81.9
Pseudolebinthus sp.	16,075	75.8	-0.111	11,187	3727	75.2	-0.315	1278	76.6	790	74.4	1342	80.1
Svistella anhuiensis	16,494	73.8	-0.06	11,180	3724	71.6	-0.286	1312	75.2	797	74.9	1416	84.6

Tarbinskiellus portentosus	15,498	72.8	-0.116	11,203	3733	72.2	-0.32	1289	74.9	816	70.2	736	77.5
Tarbinskiellus sp. *	15,514	72.8	-0.115	11,200	3732	72.2	-0.32	1290	74.7	767	70		
Teleogryllus infernalis	15,512	73.9	-0.083	11,166	3720	73.3	-0.289	1299	76.7	812	72.8	759	77
*Teleogryllus occipitalis**	15,501	73.5	-0.095	11,210	3735	73	-0.285	1286	74.9	764	71.5		
Trigonidium sjostedti	15,763	76.9	-0.02	11,201	3733	75.9	-0.193	1297	77.9	1004	78.5	498	79.3
*Truljalia hibinonis**	15,120	75.4	-0.068	11,142	3713	74.6	-0.236	1269	75.3	789	76.6		
Turanogryllus eous	16,045	71.1	-0.127	11,177	3722	70	-0.312	1310	74.7	778	71.9	1292	72.4
Velarifictorus hemelytrus	16,123	72.6	-0.093	11,179	3724	71.7	-0.29	1315	75.7	805	72.7	1396	73.4
Xenogryllus marmoratus	15,762	72.1	-0.132	11,198	3731	70.8	-0.34	1285	74.7	759	70.5	711	80.3
Meloimorpha japonica	15,880	72.5	-0.073	11,215	3737	71.7	-0.25	1271	74.8	756	72.9	1180	74
Loxoblemmus equestris	16,314	71.9	-0.122	11,178	3724	71.2	-0.302	1289	74.6	766	70.8	1592	71.6
Homoeoxipha nigripes	15,679	77.6	-0.028	11,169	3721	76.4	-0.226	1285	79.1	759	78.8	644	86.8
Gryllus veletis	15,686	73.6	-0.089	11,207	3735	73.5	-0.298	1284	76.3	804	72	952	67.5
Gryllus lineaticeps	15,607	72.6	-0.1	11,217	3738	72.8	-0.31	1324	75.8	813	67.3	810	83.8
*Gryllus bimaculatus**	16,075	74.1	-0.089	11,208	3735	73.6	-0.297	1321	75.1	743	74.3		
Gryllodes sp. *	15,550	70.6	-0.075	11,186	3727	70.1	-0.282	1331	73.4	760	68.7		
Gryllodes sigillatus	16,369	70.4	-0.075	11,183	3726	70.1	-0.281	1310	73.5	760	68.7	1653	67.6
Dianemobius furumagiensis	15,350	73.8	-0.014	11,138	3710	72.9	-0.165	1302	75.9	769	72.2	519	81.1
Dianemobius fascipes	15,363	72.3	-0.01	11,136	3709	71.3	-0.165	1306	73.3	763	70.5	558	81.9
Cardiodactylus muiri	16,328	76.5	-0.083	11,207	3734	75.5	-0.279	1312	78.7	771	74.9	1472	80.1
Cacoplistes rogenhoferi	16,018	73.3	-0.068	11,203	3733	73	-0.228	1278	75.1	763	73	1264	72

Note: * the mitochondrial genome of the indicated species is incomplete.

Table S3 Codon usage analysis of *Schizodactylus jimo* mitogenomes

Codon	Count	RSCU	Codon	Count	RSCU	Codon	Count	RSCU	Codon	Count	RSCU
UUU(F)	165	1.14	UCU(S)	66	1.54	UAU(Y)	109	1.34	UGU(C)	33	1.47
UUC(F)	125	0.86	UCC(S)	59	1.38	UAC(Y)	54	0.66	UGC(C)	12	0.53
UUA(L)	164	1.68	UCA(S)	81	1.89	UAA(*)	9	1.64	UGA(W)	75	1.4
UUG(L)	116	1.19	UCG(S)	13	0.3	UAG(*)	2	0.36	UGG(W)	32	0.6
CUU(L)	56	0.57	CCU(P)	38	1.06	CAU(H)	43	0.92	CGU(R)	12	0.81
CUC(L)	55	0.56	CCC(P)	53	1.48	CAC(H)	50	1.08	CGC(R)	7	0.47
CUA(L)	158	1.61	CCA(P)	45	1.26	CAA(Q)	52	1.41	CGA(R)	31	2.1
CUG(L)	38	0.39	CCG(P)	7	0.2	CAG(Q)	22	0.59	CGG(R)	9	0.61
AUU(I)	191	1.23	ACU(T)	57	1.09	AAU(N)	97	1.24	AGU(S)	35	0.82
AUC(I)	120	0.77	ACC(T)	81	1.54	AAC(N)	60	0.76	AGC(S)	16	0.37
AUA(M)	158	1.33	ACA(T)	62	1.18	AAA(K)	39	1.1	AGA(S)	59	1.38
AUG(M)	79	0.67	ACG(T)	10	0.19	AAG(K)	32	0.9	AGG(S)	14	0.33
GUU(V)	76	1.32	GCU(A)	67	1.29	GAU(D)	44	1.21	GGU(G)	61	1.03
GUC(V)	32	0.56	GCC(A)	75	1.44	GAC(D)	29	0.79	GGC(G)	35	0.59
GUA(V)	76	1.32	GCA(A)	44	0.85	GAA(E)	52	1.25	GGA(G)	74	1.25
GUG(V)	46	0.8	GCG(A)	22	0.42	GAG(E)	31	0.75	GGG(G)	66	1.12
